# CUFID-query: accurate network querying through random walk based network flow estimation

**DOI:** 10.1186/s12859-017-1899-y

**Published:** 2017-12-28

**Authors:** Hyundoo Jeong, Xiaoning Qian, Byung-Jun Yoon

**Affiliations:** 1 Department of Electrical and Computer Engineering, Texas A&M University, College Station, 77843 TX USA; 2 Department of Neuroogy, Baylor College of Medicine, Houston, TX USA; 3 Jan and Dan Duncan Neurological Research Institute, Texas Children’s Hospital, Houston, TX USA; 4 TEES-AgriLife Center for Bioinformatics and Genomic Systems Engineering (CBGSE), College Station, TX USA

**Keywords:** Comparative network analysis, Network querying, Random walk

## Abstract

**Background:**

Functional modules in biological networks consist of numerous biomolecules and their complicated interactions. Recent studies have shown that biomolecules in a functional module tend to have similar interaction patterns and that such modules are often conserved across biological networks of different species. As a result, such conserved functional modules can be identified through comparative analysis of biological networks.

**Results:**

In this work, we propose a novel network querying algorithm based on the CUFID (Comparative network analysis Using the steady-state network Flow to IDentify orthologous proteins) framework combined with an efficient seed-and-extension approach. The proposed algorithm, CUFID-query, can accurately detect conserved functional modules as small subnetworks in the target network that are expected to perform similar functions to the given query functional module. The CUFID framework was recently developed for probabilistic pairwise global comparison of biological networks, and it has been applied to pairwise global network alignment, where the framework was shown to yield accurate network alignment results. In the proposed CUFID-query algorithm, we adopt the CUFID framework and extend it for local network alignment, specifically to solve network querying problems. First, in the seed selection phase, the proposed method utilizes the CUFID framework to compare the query and the target networks and to predict the probabilistic node-to-node correspondence between the networks. Next, the algorithm selects and greedily extends the seed in the target network by iteratively adding nodes that have frequent interactions with other nodes in the seed network, in a way that the conductance of the extended network is maximally reduced. Finally, CUFID-query removes irrelevant nodes from the querying results based on the personalized PageRank vector for the induced network that includes the fully extended network and its neighboring nodes.

**Conclusions:**

Through extensive performance evaluation based on biological networks with known functional modules, we show that CUFID-query outperforms the existing state-of-the-art algorithms in terms of prediction accuracy and biological significance of the predictions.

## Background

Proteins have their own functions and identifying their functions is a critical step to decipher underlying biological mechanisms in a cell. In addition to investigating functions of an individual protein, taking a set of proteins and their interactions into account is significantly effective to identify novel functions of proteins because particular protein-protein interactions (PPI) can inhibit or promote a certain biological process. These proteins and their interactions can form a functional module that performs a particular biological function, and identifying functional modules is necessary to understand core biological mechanisms in a cell and it can be utilized to design a novel drug, effective diagnosis, and therapy for complex disease such as cancer [1–3].

The recent advances in high-throughput profiling techniques have enabled systematic analysis of protein interactions, providing abundant prior knowledge for PPI networks. For instance, as novel protein interactions corresponding to a specific regulatory process have been identified, the number of known functional modules have been gradually increasing. However, biological experiments to identify such functional modules still require a huge amount of resources such as labor, cost, and time. As it has been proved that functional modules or signaling pathways are often well conserved across different biological networks [4, 5], comparative network analysis has been emerging computational means to identify and predict conserved functional modules in different biological networks [6].

Network querying is one of comparative network analysis methods, where it aims to search the large-scale target network and determine whether the target network includes the subnetwork that are similar to the given query network such as signaling pathways or functional modules in terms of biological functions. Through a network querying algorithm, we can identify the conserved functional modules and predict the functions of the conserved network in the target network based on the functions of the query (i.e., transferring the prior knowledge of the well-studied species to the under-studied species). Additionally, network querying can be utilized to predict novel functional modules.

Several network querying algorithms have been proposed [7–16]. PathBLAST [7] is one of pioneering network querying algorithms, but it can search only linear pathways and the computational complexity limits the size of the query network. QPath [8] can search much longer pathways than PathBLAST and QNet [9] can search both linear pathways and tree structure, but both algorithms still require high computational complexity and searching capability is limited to either a pathway or a tree. PathMatch [10] solves a network querying problem by finding the longest weighted path in a directed acyclic graph (target network) and GraphMatch [10] finds highest scoring subgraphs in a target network using an exact algorithm. SAGA [11] solves an approximated graph matching based on the fragment index, where it is the index on a small substructure of graphs in a database, and SAGA employs a flexible model for node gaps/mismatches and network structural variations. NatalieQ [12] identifies the querying results by solving the integer linear programming through Lagrangian relaxation combined with a branch-and-bound approach. TORQUE [13] proposed a topology-free network querying algorithm. That is, it only requires a set of proteins in the query network and it does not necessary to provide the topological structure of the query network. TORQUE finds a connected set of matching proteins through a dynamic and integer linear programming based on a sequence similarity of proteins. RESQUE [14] estimates the node-to-node correspondence through a semi-Markov random walk (SMRW) model [17]. Then, RESQUE iteratively removes less relevant nodes in the target network and identifies the best matching subnetwork through either a Hungarian method or identifying the largely connected subnetwork. Corbi [15] estimates a matching probability of nodes in the query and target network through a conditional random field (CRF), and identifies the matching subnetwork through iterative bi-directional mapping. SEQUOIA [16] adopts the context-sensitive random walk model [18] to estimate the node correspondence scores and adopts seed-and-extension approach to identify the conserved networks.

**Fig. 1:**
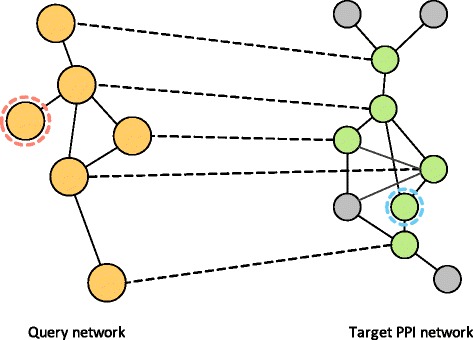
Illustration of a typical network querying problem. The node marked in red in the query network is deleted in the target network, and the node marked in blue is not present in the query but inserted in the target network. Note that the terms insertion/deletion are relative, and an inserted node in one network can be viewed as a deleted node in the other network

In this paper, we propose CUFID-query, a novel network querying algorithm to identify the conserved subnetwork in the target PPI network that considers both molecular and topological/structural properties. The proposed network querying algorithm addresses two major challenges in a network querying problem: high computational complexity and structural variation of the conserved networks. To tackle the complexity issue, we adopt the CUFID (**C**omparative network analysis **U**sing the steady-state network **F**low to **ID**entify orthologous proteins) framework [19], where it is originally designed to estimate node-to-node correspondence for large-scale biological networks. Typically, as a network querying algorithm requires to examine a large-scale target network in order to find the best matching subnetwork that is similar to the given small query network, computationally efficient method to scan a large searching space is necessary. Additionally, we utilize a seed-and-extension approach in order to deal with structural variations of conserved networks. As illustrated in Fig. 1, although the conserved subnetwork performs the similar biological functions, there are inserted and deleted nodes and edges, and these structural changes make it difficult to solve a network querying problem through a classical bipartite matching problem.

In the proposed method, we first estimate the node-to-node correspondence (i.e., biological relevance or matching probability) between query and target networks. Then, based on the estimated node correspondence scores, we select the largely connected seed network through a maximum weighted bipartite matching algorithm. Next, we iteratively extend the seed network by including the node that meets the following two conditions: 1) larger association probability and 2) minimizing a conductance of the extending seed network from the rest of the target network. The association probability could estimate the frequency of interactions between the nodes in the extending seed network and the neighboring nodes of the seed network. Including the neighboring node with more interactions to the nodes in the seed network can be advantageous to lead functionally consistent querying results because proteins having a direct interaction have more chances to share and perform the similar biological functions [20]. Note that since we only consider the nodes in the target network as a candidate for a network extension, the searching space for the network extension is limited to the nodes in the target network. In the extension step, we list all candidate nodes based on the association probability and select the winning node that can maximally minimize the conductance of the extending seed network. This rule selects the node having a higher probability to frequently interact with the nodes in the extending seed network as well as rarely interact to the rest of the network. Finally, after completing the extension steps, we remove less relevant nodes in the fully extended network based on the personalized PageRank vector [21] in order to increase the functional consistency of the querying result.

## Methods

### Problem formulation and overview of the proposed method

Suppose that we have a query network and it can be represented as a graph ${\mathcal {G}}_{\mathcal {Q}} = \left ({{\mathcal {V}}_{\mathcal {Q}},{\mathcal {E}}_{\mathcal {Q}}} \right)$. For example, a node $v_{i} \in {\mathcal {V}}_{\mathcal {Q}} $ indicates a protein in the query network and an edge $e_{i,j} \in {\mathcal {E}}_{\mathcal {Q}} $ represents the interaction or binding between protein *v*_*i*_ and protein *v*_*j*_. In this study, since we consider protein-protein interactions networks, we limit our scope to undirected networks. When two proteins *v*_*i*_ and *v*_*j*_ interact with each other, hence connected by an edge *e*_*i*,*j*_ in the network, it is assumed that there is a directional edge from *v*_*i*_ to *v*_*j*_ and also from *v*_*j*_ to *v*_*i*_. Similarly, suppose that a target network is given and represented by a graph ${\mathcal {G}}_{\mathcal {T}} = \left ({{\mathcal {V}}_{\mathcal {T}},{\mathcal {E}}_{\mathcal {T}}} \right)$. We assume that a pairwise node similarity score *s*(*v*_*q*_,*v*_*t*_) is given for $\forall v_{q} \in {\mathcal {V}}_{\mathcal {Q}} $ and $\forall v_{t} \in {\mathcal {V}}_{\mathcal {T}} $, where it is proportional to the molecular level similarity of two proteins (*v*_*q*_,*v*_*t*_). In this study, we considered protein-protein interactions (PPI) networks, and we utilized BLAST bit scores as pairwise node similarity scores but other types of similarity measurements or their combination can be utilized. Generally, in a network querying problem, the size of the target network is significantly larger than the size of the query network, i.e., $\left | {{\mathcal {V}}_{\mathcal {Q}}} \right | \ll \left | {{\mathcal {V}}_{\mathcal {T}}} \right |$, where the size of the network is the number of nodes in the network.

The goal of network querying is to identify the conserved subnetworks that is expected to perform the same or similar biological functions to the given query network. Hence, the network querying problem is formulated as the following optimization problem:

1 $$ \hat {\mathcal{G}}_{\mathcal{T}}^{*} = \underset{{\forall} \hat {\mathcal{G}}_{\mathcal{T}} \in \mathbf{G}_{\mathbf{T}}}{\arg \max}\ f\left({\hat {\mathcal{G}}_{\mathcal{T}},{\mathcal{G}}_{\mathcal{Q}}} \right),   $$

where **G**_**T**_ is a feasible set of all subnetworks in the target PPI network, and $f\left ({ {\mathcal {G}}_{\mathcal {X}},{\mathcal {G}}_{\mathcal {Y}}} \right)$ is the function that can quantitatively estimate the functional similarity or biological relevance of two biological networks $\left ({ {\mathcal {G}}_{\mathcal {X}},{\mathcal {G}}_{\mathcal {Y}}} \right)$.

Network querying can be viewed as a subgraph isomorphism problem, where it determines whether one graph (query network) is isomorphic to the subgraph of the target graph (target PPI network). Solving the network querying problem as the subgraph isomorphism problem, considering possible node (or edge) insertion and deletion in each network, is NP-complete [22]. Additionally, identifying the conserved subnetwork in the target network is practically difficult because of the following reasons: 1) it is not straightforward to compute node correspondence scores as the scale of the biological network is very large (i.e., complexity problem), 2) quantitatively estimating the functional similarity $f\left ({ {\mathcal {G}}_{\mathcal {X}},{\mathcal {G}}_{\mathcal {Y}}} \right)$ of two biological networks is difficult, and 3) we have no prior knowledge whether the size of the conserved subnetwork is larger or smaller than the query network because of the structural variations in biological networks. That is, we have no prior knowledge for the exact number of inserted/deleted nodes.

To overcome these challenges, we propose a heuristic network querying algorithm based on the CUFID framework and a seed-and-extension approach. In the proposed network querying algorithm called CUFID-query, we first compute the node-to-node correspondence scores through the CUFID framework. The CUFID framework can effectively deal with the complexity problem as it can estimate the node correspondences for large-scale networks with a low computational cost. Based on the intuition that two proteins in different networks would be an orthologous pair if they have a high molecular similarity as well as the similar interaction patterns to its neighboring nodes [4, 23], the CUFID framework can effectively estimate a biological relevance between the nodes in the query and target network by integrating the molecular and topological similarities in a balanced manner. After computing the node correspondence scores, we induce a seed network using the seed nodes that can be identified through a maximum weighted bipartite matching algorithm. Note that the seed network ${\mathcal {G}}_{\mathcal {S}}=\left ({\mathcal {V}}_{\mathcal {S}}, {\mathcal {E}}_{\mathcal {S}} \right)$ is always smaller than the query network (i.e., $\left | {\mathcal {V}}_{\mathcal {S}} \right | \leq \left | {\mathcal {V}}_{\mathcal {Q}} \right |$). Then, we iteratively extend the seed network using a probabilistic framework, where it is designed to select the nodes that can have more interactions to the nodes in the seed network and minimize the conductance of the extending seed network from the rest of the target network. Finally, we removed less relevant nodes based on the personalized PageRank vector. Due to the structural variations between conserved functional modules, solving a subgraph isomorphic problem may not the best way to find the solution to a network querying problem in a practical point of view, and a seed-and-extension approach could be a reasonable alternative. However, since the approach is not the optimal and less relevant nodes could be included in the network extension steps, effective post-processing to remove less relevant nodes can increase the accuracy of a querying result.

### Estimating the node correspondence through the CUFID framework

The proposed network querying algorithm adopts a seed-and-extension approach to efficiently deal with the structural variations of functional modules that are conserved in different biological networks. We utilize the CUFID framework to select the seed nodes in the target network that have high correspondence to the query nodes. We recently proposed the CUFID framework for global comparison and alignment of large-scale biological networks with comparable number of nodes. However, we show in this study that the framework is also effective for estimating the node correspondence for biological networks with significantly different sizes.

In the following, we first briefly review the CUFID framework that can be utilized to estimate the node-to-node correspondence between the query and target networks. As shown in [4, 23], taking the node and topological similarities into account can lead to an improved prediction accuracy when comparing different biological networks and identifying orthologous proteins. Here, the node similarity indicates the molecular similarity of the proteins, and the topological similarity denotes the likeness of the interaction patterns to its neighboring nodes. Hence, if the molecular composition of two proteins in different biological networks are highly similar each other and there are more number of neighboring nodes with a close composition, they are highly likely to be orthologous (i.e., performing the same or similar biological functions). Recently, random walk based approaches have been successfully applied to integrate both node and topological similarities [14, 16–19, 23–26] because of its distinctive advantages: First, a network comparison based on a random walk model is flexibility for topological structures of networks (i.e., it can estimate node correspondences for any network topology such as pathway, tree, and clique). It is also robust to structural variations such as node insertions/deletions. Furthermore, random walk based approaches are typically computationally efficient because the real-world biological networks generally have sparse interactions. For example, IsoRank [23] and IsoRankN [24] adopt the Google’s PageRank algorithm [27] in order to estimate node correspondence scores. It performs a random walk over the Kronecker product between two networks, and utilizes the score to derive a global network alignment. SMETANA [25] and RESQUE [14] adopt a semi-Markov random walk (SMRW) model [17]. In the SMRW model, the staying time of the random walker at the pair of nodes is proportional to the topological similarity and the pairwise node similarity between networks. In the context-sensitive random walk (CSRW) model [18], the next position of the random walker is determined by the similarity of the neighboring nodes at the current position of the random walker (i.e., context of the current position of the random walker), and it can accurately estimate the node correspondences by dealing with the node insertions/deletions [16, 26]. The CUFID framework has been recently proposed to estimate the node correspondence (steady-state network flow) by effectively integrating both molecular and topological similarities using a Markov random walk model, and helped solve a pairwise global network alignment problem [19].

**Fig. 2:**
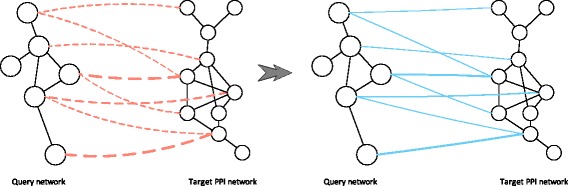
Illustration for constructing the integrated network by combining the query and target networks. Dotted lines indicate positive node similarity scores between pairs of nodes, where the thickness of each line is proportional to the similarity score. We insert a pseudo-edge between a node in the query network and a node in the target network if the corresponding proteins have a positive node similarity score

First, to estimate the node correspondence through the CUFID framework, we construct the integrated network by combining networks to be compared. Specifically, as shown in Fig. 2, we insert the pseudo-edges connecting nodes in the query and target networks if their pairwise node similarity score is greater than a threshold *s*_*t*_. That is, the integrated network can be represented as a graph ${\mathcal {G}} = \left ({{\mathcal {V}},{\mathcal {E}}} \right)$, where ${\mathcal {V}}$ denotes the union of the nodes in the query and target networks (i.e. ${\mathcal {V}} = \left \{{{\mathcal {V}}_{\mathcal {Q}}, {\mathcal {V}}_{\mathcal {T}}} \right \}$); ${\mathcal {E}}$ is the union of the edges in the two networks; and the inserted pseudo-edges such that ${\mathcal {E}}_{\mathcal {P}} = \left \{{e_{i,j} |v_{i} \in {\mathcal {V}}_{\mathcal {Q}},v_{j} \in {\mathcal {V}}_{\mathcal {T}},s\left ({v_{i},v_{j}} \right) > s_{t}} \right \}$, (i.e., ${\mathcal {E}} = \left \{ {{\mathcal {E}}_{\mathcal {Q}}, {\mathcal {E}}_{\mathcal {T}} }, {{\mathcal {E}}_{\mathcal {P}}} \right \}$). Then, we allow a random walker to transit within and across the networks to be compared.

If the random walker performs a random movement over the edges representing protein-protein interactions, it can move to its neighboring nodes belonging to the same PPI network. That is, at the current position of the random walker, it can transit to its neighboring nodes only if they are connected through the edges either ${\mathcal {E}}_{\mathcal {Q}}$ or ${\mathcal {E}}_{\mathcal {T}}$ indicating the protein-protein interactions. The transition probability for the random walk within either the query or target PPI networks is given by

2 $$ \mathbf{P}_{\mathbf{Q}} = \mathbf{D}_{\mathbf{Q}}^{- 1} \cdot \mathbf{A}_{\mathbf{Q}}\,\, \text{and}\,\, \mathbf{P}_{\mathbf{T}} = \mathbf{D}_{\mathbf{T}}^{- 1} \cdot \mathbf{A}_{\mathbf{T}},   $$

where **A**_**Q**_ (or **A**_**T**_) is an adjacency matrix of the query (or target) network and **D**_**Q**_ (or **D**_**T**_) is a diagonal matrix such that ${{D}}_{{Q}}\left [ {i,i} \right ] = \sum \limits _{\forall j} {{{A}}_{{Q}} \left [ {i,j} \right ]} $ (or ${{D}}_{{T}}\left [ {i,i} \right ] = \sum \limits _{\forall j} {{{A}}_{{T}} \left [ {i,j} \right ]} $).

The random walker can also transit across the query and target networks through the pseudo-edges ${ {\mathcal {E}}_{\mathcal {P}}} $. When the random walker transits from the query network to the target PPI network, the transition probability of the random walker for this event is given by

3 $$ \mathbf{P}_{\mathbf{Q} \to \mathbf{T}} = \mathbf{D}_{\mathbf{S}}^{- 1} \cdot \mathbf{S},   $$

where **S** is a $\left | {{\mathcal {V}}_{\mathcal {Q}}} \right | \times \left | {{\mathcal {V}}_{\mathcal {T}}} \right |$ dimensional matrix for the pairwise node similarity score such that $S\left [ i,j \right ] = s\left (v_{i}, v_{j}\right), \forall v_{i} \in {\mathcal {V}}_{\mathcal {Q}},\forall v_{j} \in {\mathcal {V}}_{\mathcal {T}}$, and **D**_**S**_ is a $\left | {{\mathcal {V}}_{\mathcal {Q}}} \right | \times \left | {{\mathcal {V}}_{\mathcal {Q}}} \right |$ dimensional diagonal matrix such that ${{D}}_{{S}}\left [ {i,i} \right ] = \sum \limits _{\forall j} {{{S}} \left [ {i,j} \right ]} $.

Similarly, if the random walker jumps from the target PPI network to the query network, the transition probability is given by

4 $$ \mathbf{P}_{\mathbf{T} \to \mathbf{Q}} = \mathbf{S}^{\mathrm{T}} \cdot \mathbf{D}_{\mathbf{S}^{\mathrm{T}} }^{- 1}.   $$

We can construct the overall transition probability matrix for the random walker over the integrated network ${\mathcal {G}}$ by concatenating the above probability matrices as follows:

5 $$ \mathbf{P} = \left[{\begin{array}{ll} {\mathbf{P}_{\mathbf{Q}}} & {\mathbf{P}_{\mathbf{Q} \to \mathbf{T}}} \\ {\mathbf{P}_{\mathbf{T} \to \mathbf{Q}}} & {\mathbf{P}_{\mathbf{T}}} \\ \end{array}} \right],   $$

with necessary normalization to make the matrix **P** a stochastic matrix. We can compute the corresponding steady-state probability *π* of the random walker, where it is equivalent to the expected time of the random walker staying at the particular node in long term. Since real PPI networks have generally sparse interactions, the steady-state probability can be easily obtained through a power method [19].

**Fig. 3:**
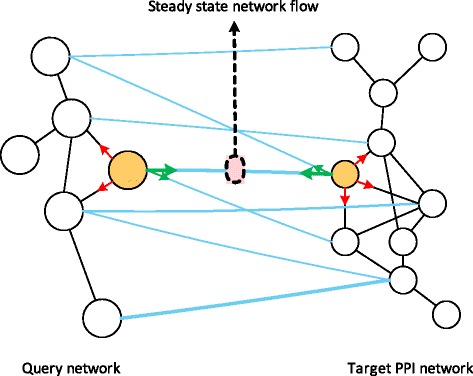
Estimating the steady-state network flow based on the CUFID framework. Red arrows indicate the random walk within the query or the target network, while the green arrows represent the random walk across two networks. The correspondence between two nodes – one in the query network and the other in the target network – can be estimated by measuring the steady-state network flow through the pseudo-edges connecting the nodes

Finally, as shown in Fig. 3, the node-to-node correspondence between the query and target networks can be obtained by estimating the steady-state network flow (i.e., traversal of the random walker) across the pseudo-edges connecting the nodes in the query and target networks, which is given by

6 $$ \mathbf{C} = \bar \pi_{\mathcal{Q}} \cdot \mathbf{P}_{\mathbf{Q} \to \mathbf{T}} + \mathbf{P}_{\mathbf{T} \to \mathbf{Q}}^{\mathrm{T}} \cdot \bar \pi_{\mathcal{T}},   $$

where $\bar \pi _{\mathcal {Q}}$ is a $\left | {{\mathcal {V}}_{\mathcal {Q}}} \right | \times \left | {{\mathcal {V}}_{\mathcal {Q}}} \right |$ dimensional diagonal matrix such that $\bar \pi _{\mathcal {Q}} \left [ {i,i} \right ] = \pi \left ({v_{i}} \right),\forall v_{i} \in {\mathcal {V}}_{\mathcal {Q}} $ and $\bar \pi _{\mathcal {T}}$ is a $\left | {{\mathcal {V}}_{\mathcal {T}}} \right | \times \left | {{\mathcal {V}}_{\mathcal {T}}} \right |$ dimensional diagonal matrix such that $\bar \pi _{\mathcal {T}} \left [ {i,i} \right ] = \pi \left ({v_{j}} \right),\forall v_{j} \in {\mathcal {V}}_{\mathcal {T}} $.

### Proposed network querying algorithm: CUFID-query

The proposed network querying algorithm – CUFID-query – has three main steps. First, we compute the node-to-node correspondence between the query and target networks through the CUFID framework. Next, we select the seed network (i.e., high scoring subnetwork) and iteratively extend the seed network in the target network until it meets the stop conditions. Finally, we remove less relevant nodes based on the personalized PageRank (PPR) vector of the induced network.

Once we obtained the node correspondence between the query and target networks through Eq. (6), we select the seed nodes by maximum weighted bipartite matching implemented in the MATLAB GAIMC toolbox [28]. Then, we construct the induced seed network based on the selected seed nodes (i.e., the matching nodes in the target network corresponding to the nodes in the query network). If the induced network is disconnected, we will use the largest connected network as the seed network. If all the seed nodes are disconnected, we will select a single node with the maximum correspondence score as the seed.

Next, we iteratively extend the seed network by adding a node based on the association probability and the network conductance minimization principle. To this aim, we define the association probability as the likelihood that the random walker starting from a node in the seed network will return to the seed network within 2 hops by passing through the neighboring nodes of the seed network. Note that, since we focus on undirected networks, if there is an edge *e*_*i*,*j*_ between two proteins *v*_*i*_ and *v*_*j*_, our random walker can move from *v*_*i*_ to *v*_*j*_ as well as from *v*_*j*_ to *v*_*i*_ without any restriction. When a neighboring node has a higher association probability, it can have more interactions to the seed network and it is more likely to share the similar biological functions to the nodes in the seed network because interacting proteins tend to share the similar biological functions [20]. To compute the association probability, we compute the initial steady-state probability $\pi _{\mathcal {S}} $ of the random walker for the seed network. Given the seed network ${{\mathcal {G}}_{\mathcal {S}}} = \left ({{\mathcal {V}}_{\mathcal {S}},{\mathcal {E}}_{\mathcal {S}}} \right)$, the steady-state probability for the node *v*_*i*_ in the seed network is given by [29]

7 $$ \pi_{\mathcal{S}} \left({v_{i}} \right) = \frac{{d\left({v_{i}} \right)}}{{\sum\limits_{v_{i} \in {\mathcal{G}}_{\mathcal{S}}} {d\left({v_{i}} \right)} }},   $$

where *d*(*v*_*i*_) is the degree of the node *v*_*i*_.

Then, for each neighboring node *v*_*n*_ such that $v_{n} = \left \{ v_{x} | v_{x} \in {\mathcal {N}}\left ({v_{i}} \right), \forall v_{i} \in {\mathcal {V}}_{\mathcal {S}} \right \}$, the probability of the random walker jumps to the neighboring node *v*_*n*_ from any node in the seed network is given by

8 $$ {\mathrm{P}}_{1} \left({v_{n}} \right) = \sum\limits_{v_{i} \in \left\{ {{\mathcal{V}}_{\mathcal{S}} \cap {\mathcal{N}}\left({v_{n}} \right)} \right\}} {\frac{{\pi_{\mathcal{S}} \left({v_{i}} \right)}}{{d\left({v_{i}} \right)}}},   $$

where ${\mathcal {N}}\left ({v_{x}} \right)$ is the neighboring nodes of the node *v*_*x*_.

Finally, the association probability for the neighboring node *v*_*n*_ is given by

9 $$ {\mathrm{P}}_{2} \left({v_{n}} \right) = {\mathrm{P}}_{1} \left({v_{n}} \right) \cdot \frac{{r\left({v_{n}} \right)}}{{d\left({v_{n}} \right)}},  $$

where *r*(*v*_*n*_) is the number of edges connecting *v*_*n*_ and the nodes in the seed network (i.e., $\left | {\left \{ {e_{n,j} |v_{n}, v_{j} \in {\mathcal {G}}_{\mathcal {S}}} \right \}} \right |$).

We select top *K* candidate nodes having the highest association probability, and select the finalist to be included to extend the seed network based on the conductance minimization criterion. Conductance minimization criterion has been widely utilized in the non-comparative network analysis algorithms [29, 30] because proteins in the functional module typically tend to be densely connected to each other while sparsely connected to the rest of the network. Given a subnetwork ${\mathcal {G}}_{\mathcal {S}}$ in the target network (i.e., ${\mathcal {G}}_{\mathcal {S}} \subset {\mathcal {G}}_{\mathcal {T}}$), the conductance of the subnetwork is given by [21]

10 $$ \varphi \left({{\mathcal{G}}_{\mathcal{S}}} \right) = \frac{{\left| {\left\{ {e_{i,j} |v_{i} \in {\mathcal{V}}_{\mathcal{Q}},v_{j} \in {\mathcal{V}}\backslash {\mathcal{V}}_{\mathcal{Q}}} \right\}} \right|}}{{\min \left({vol\left({{\mathcal{G}}_{\mathcal{S}}} \right),2m - vol\left({{\mathcal{G}}_{\mathcal{S}}} \right)} \right)}},   $$

where *m* is the number of undirected edges and $vol\left ({\mathcal {G}} \right) = \sum \limits _{v_{i} \in {\mathcal {G}}} {d\left ({v_{i}} \right)} $. Note that the conductance defined in Eq. (10) is applicable only for undirected networks. Since the conserved subnetwork is typically much smaller than the target network (i.e., ${\mathcal {G}}_{\mathcal {S}} \ll {\mathcal {G}}_{\mathcal {T}}$), Eq. (10) becomes

11 $$ \begin{aligned} \varphi \left({{\mathcal{G}}_{\mathcal{S}}} \right) &= \frac{{\left| {\left\{ {e_{i,j} |v_{i} \in {\mathcal{V}}_{\mathcal{Q}},v_{j} \in {\mathcal{V}}\backslash {\mathcal{V}}_{\mathcal{Q}}} \right\}} \right|}}{{vol\left({{\mathcal{G}}_{\mathcal{S}}} \right)}} \\ &= \frac{{\left| {\left\{ {e_{i,j} |v_{i} \in {\mathcal{V}}_{\mathcal{Q}},v_{j} \in {\mathcal{V}}\backslash {\mathcal{V}}_{\mathcal{Q}}} \right\}} \right|}}{{\left| {\left\{ {e_{i,j} |v_{i} \in {\mathcal{V}}_{\mathcal{Q}},v_{j} \in {\mathcal{V}}_{\mathcal{Q}}} \right\}} \right|}}. \end{aligned}  $$

In the extension steps, we first select the top 20 nodes with the highest association probability, and we finally select one node that can maximally minimize the conductance of the seed network. We iteratively extend the seed network until either one of the following stopping conditions is satisfied: 1) the size of the extending seed network exceeds the limits; 2) there are no neighboring nodes that can decrease the conductance of the extending network more than 10%.

Once the seed network is fully grown, we finally refine the extended seed network by removing the less relevant nodes based on the personalized PageRank (PPR) vector. For this purpose, we construct the induced network ${\mathcal {G}}_{\mathcal {I}}$ based on the extended seed network and its neighboring nodes (i.e., ${\mathcal {G}}_{\mathcal {I}} = \left ({{\mathcal {V}}_{\mathcal {I}},{\mathcal {E}}_{\mathcal {I}}} \right)$, where ${{\mathcal {V}}_{\mathcal {I}}} = \left \{ {{\mathcal {V}}_{\mathcal {S}},{\mathcal {N}}\left ({{\mathcal {V}}_{\mathcal {S}}} \right)} \right \}$ and ${\mathcal {E}}_{\mathcal {I}} = \left \{ {{\mathcal {E}}_{\mathcal {S}},{\mathcal {E}}_{\mathcal {A}}} \right \}$ such that ${\mathcal {E}}_{\mathcal {A}} = \left \{ {e_{i,j} |v_{i} \in {\mathcal {V}}_{\mathcal {S}},v_{j} \in {\mathcal {N}}\left ({{\mathcal {V}}_{\mathcal {S}}} \right)} \right \}$). Then, we compute the PPR vector for the induced network ${\mathcal {G}}_{\mathcal {I}} $. The standard PPR vector **r** is a unique solution of the following equation: [21]

12 $$ \mathbf{r} = \alpha \cdot \mathbf{s} + \left({1 - \alpha} \right) \cdot \mathbf{r} \cdot \mathbf{M},   $$

where *α* is a teleportation constant and we set *α* as 0.5, **M** is the normalized adjacency matrix of the induced network ${\mathcal {G}}_{\mathcal {I}}$ and **s** is a preference vector. We set the preference vector **s** as follows:

$$s\left({v_{i}} \right) = \left\{ \begin{array}{ll} 1 \left/ \left| \mathcal{V}_{\mathcal{S}} \right|\right., & {v_{i} \in {\mathcal{V}}_{\mathcal{S}}} \\ {0,} & {otherwise.} \end{array} \right. $$

Once we obtain the PPR vector for the induced network ${\mathcal {G}}_{\mathcal {I}}$, we iteratively select the nodes with the highest PPR vector values until the cumulative sum becomes 0.5. In this pruning step, it would be possible that the nodes in the extended seed network could be removed and other neighboring nodes would be included in the final querying results. Note that this pruning process could make the querying results disconnected. If the identified network is fragmented by the pruning step, CUFID-query only returns the largest connected network as the querying results. The steps of CUFID-query are summarized in Algorithm 1. We briefly compare SEQUOIA [16] and CUFID-query as they both adopt similar seed-and-extension approaches. One important difference between the seed extension steps in the two algorithms is that SEQUOIA extends the intermediate networks only based on the conductance minimization principle while CUFID-query adopts the conductance minimization principle and simultaneously uses the association probability to select additional nodes. Furthermore, in the post-processing step, SEQOIA only removes irrelevant nodes in the extended seed network, but CUFID-query can recruit new nodes that are originally not included in the extended seed network by utilizing the PPR vector of the induced network ${\mathcal {G}}_{\mathcal {I}}$.



## Results and discussion

### Datasets and experimental set-up

To assess the performance of CUFID-query, we performed experiments based on the known biological complexes and real-world PPI networks for three species: *H. sapiens* (human), *S. cerevisiae* (yeast), and *D. melanogaster* (fly). We obtained target PPI networks from STRING v10 [31]. Then, we extracted the protein-protein interactions classified as a ‘binding’ (direct interaction) and removed the protein-protein interactions without an experimental validation. We further removed protein-protein interactions with the confidence score less than 400 that indicates a medium level confidence. After the aforementioned pre-processing, the human PPI network includes 12,049 proteins and 95,209 interactions, the mouse PPI network includes 10,428 proteins and 112,541 interactions, and the yeast PPI network includes 5726 proteins and 88,308 interactions. To obtain the pairwise node similarity score for each network pair, we computed BLAST bit scores between amino acids sequences for each protein pair through BLAST version 2.3. Note that the amino acid sequences for each species were obtained from STRING v10.

We obtained the known biological complexes for human and mouse from CORUM [32], and known biological complexes for yeast are obtained from SGD [33] (accessed at Feb. 1 2017). Then, we extracted the connected networks with the size of 4 to 25. We obtained overall 1242 test cases, where the 371 human complexes were queried against the mouse PPI network, the 349 human complexes were queried against the yeast PPI network, the 64 mouse complexes were queried against the human PPI network, the 54 mouse complexes were queried against the yeast PPI network, the 201 yeast complexes were queried against the human PPI network, the 203 yeast complexes were queried against the mouse PPI network.

To assess the biological significance of the querying results, we performed a GO enrichment test for the querying results. To this aim, we downloaded the GO ontology and annotation files for each species from Gene Ontology Consortium [34] (accessed at Feb. 2 2017), and we only used GO terms with the following experimental evidence codes: ‘EXP’, ‘IDA’, ‘IPI’, ‘IMP’, ‘IGI’, and ‘IEP’. Additionally, we retained GO terms whose information contents (IC) is greater than 2 in order to perform GO enrichment test based on the more informative terms as recommended in [34]. IC is given by

13 $$ IC\left(g \right) = - \log_{2} \frac{{\left| g \right|}}{{\left| {root\left(g \right)} \right|}},   $$

where |*g*| is the number of proteins that are annotated with the particular GO term *g*, and |*r* *o* *o* *t*(*g*)| is the number of proteins belonging to the root GO term of the GO term *g*. Note that, due to the hierarchical structure, every GO term belongs to one of the root terms: biological process (BP, GO:0008150), cellular component (CC, GO:0005575), and molecular function (MF, GO:0003674). We used the latest version of GO::TermFinder [35] to perform the GO enrichment test for the querying results.

We compared the performance of CUFID-query against state-of-the-art algorithms: NatalieQ [12], Corbi [15], RESQUE [14], SEQUOIA [16] and HubAlign [36]. We used default parameters for NatalieQ. In the R package for Corbi, we used a function for a network querying with the default parameters and set the query type as a general querying because we cannot get the results when we set the query type as a heuristic querying. Although HubAlign is a pairwise network alignment algorithm, we used HubAlign to compare the performance of network querying algorithm because network querying can be classified as a special case of a local network alignment.

### Performance assessment metrics

There is no gold standard benchmark for the network querying problem. Moreover, since the exact node-to-node mapping between conserved biological complexes is also unknown, we cannot compute general performance metrics such as precision and recall for network querying algorithms. To assess the performance of the querying algorithms, we defined various performance metrics. First, since network querying algorithms can be utilized to predict novel biological complexes, we performed GO enrichment test for the querying results through GO::TermFinder [35], and if the false discovery rate (FDR) corrected *p*-value of the querying result is smaller than 0.01, we considered that the querying result is biologically significant so that it has a potential to be a functional module. Then, we counted the number of hits, defined as the querying results whose FDR corrected *p*-values are smaller than 0.01. Among these hits, we also counted the number of meaningful hits that are connected querying results whose FDR corrected *p*-value is smaller than 0.01.

Next, we defined a specific hit as the querying result that is highly overlapped with the know biological complexes. To determine whether the querying result is well-matched to the known biological complexes, we computed the match score of the querying result by comparing it to the known biological complexes ${\mathcal {R}} = \left \{ {{\mathcal {G}}_{1},{\mathcal {G}}_{1},\ldots,{\mathcal {G}}_{N}} \right \}$. Given two biological complexes ${\mathcal {G}}_{\mathcal {X}}$ and ${\mathcal {G}}_{\mathcal {Y}}$, the matching score is a Jaccard similarity index, which is given by [37]

14 $$ match\_score\left({{\mathcal{G}}_{\mathcal{X}},{\mathcal{G}}_{\mathcal{Y}}} \right) = \frac{{\left| {{\mathcal{V}}_{\mathcal{X}} \cap {\mathcal{V}}_{\mathcal{Y}}} \right|}}{{\left| {{\mathcal{V}}_{\mathcal{X}} \cup {\mathcal{V}}_{\mathcal{Y}}} \right|}}.   $$

Given a querying result ${\mathcal {G}}_{{\mathcal {Q}}^{*}} $, we computed the match score $match\_score\left ({{\mathcal {G}}_{{\mathcal {Q}}^{*}},{\mathcal {G}}_{\mathcal {X}}} \right)$ for all ${\mathcal {G}}_{\mathcal {X}} $ in ${\mathcal {R}}$, and if there is at least one known complex that yields the match score greater than a threshold *m*_*t*_, we considered the query result as a specific hit. In this study, we used a threshold *m*_*t*_ of 0.5 as in [37].

We also checked the specificity of the querying results because a querying result may contain irrelevant nodes even though it can detect the functional modules. Querying results including many irrelevant nodes may decrease the reliability of the querying algorithm, and it may not be appropriate in practical applications as it requires additional biological experiments for validation. To this aim, a specificity was defined as the ratio of the number of annotated nodes to the overall number of nodes in the querying result. In this experiment, we selected the enriched GO term with the smallest FDR corrected *p*-value, and counted the number of nodes annotated with the GO term.

Finally, we also compared the running time of each method in order to compare the computational complexity.

### Performance assessment: hits and meaningful hits

**Fig. 4:**
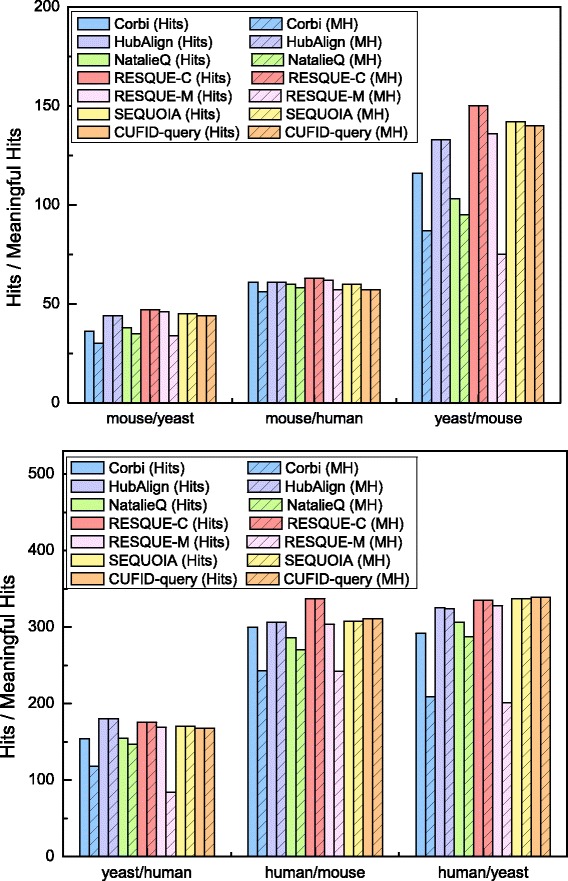
The number of hits and the number of meaningful hits are shown for each network querying algorithm. The bars shown in solid colors indicate hits and the shaded bar indicate meaningful hits. Labels in the horizontal axis show the (query species)/(target PPI network) pairs

Figure 4 shows the number of hits and meaningful hits for all the query and target pairs. As shown in Fig. 4, although RESQUE-C can identify a slightly larger number of hits, CUFID-query achieves a comparable number of hits to the other methods. CUFID-query, SEQUOIA, HubAlign, and RESQUE-M show the similar performances in terms of hits. Among six methods, the sizes of the querying results for RESQUE-C are mostly larger than those of other methods. Including more proteins in the query results can lead to more enriched GO terms with biological significance because biological complexes can be overlapped and proteins can perform multiple functions. As a result, RESQUE-C has a higher chance to achieve a higher number of hits than the other methods. Although RESQUE-C achieves the largest number of hits, we will show later that RESQUE-C includes a larger number of irrelevant nodes in the querying results that can decrease the specificity of the querying results. HubAlign and RESQUE-M show the comparable performance to CUFID-query, but we will also present that they can identify a relatively smaller number of annotated nodes. When considering one of goals for network querying, predicting and annotating functions of proteins in the target network based on the functions of the query network, identifying more annotated proteins is much advantageous. Results in Fig. 4 implies that CUFID-query has a strong potential to identify a novel functional module conserved in the target PPI network.

Next, when considering meaningful hits, CUFID-query outperforms Corbi, NatalieQ, and RESQUE-M for all query-target pairs by achieving 52, 42, and 18% more meaningful hits, respectively. Although RESQUE-M records a similar number of hits to CUFID-query, the number of meaningful hits is much smaller than that of CUFID-query because RESQUE-M does not guarantee the connected querying results. Similarly, Corbi and NatalieQ may also identify disconnected subnetworks as their querying results, which can decrease the number of meaningful hits. Identifying a connected querying result is practically important because interactions between proteins can trigger or inhibit a particular cellular mechanism and disconnected querying results may not be helpful to decipher and interpret the functions of proteins and their relationships. That is, achieving a higher number of meaningful hits instead of any hits is more important in practice. Based on these results, CUFID-query is advantageous to identify and predict protein-protein interactions that cause particular biological processes.

### Performance assessment: specific hits

**Fig. 5:**
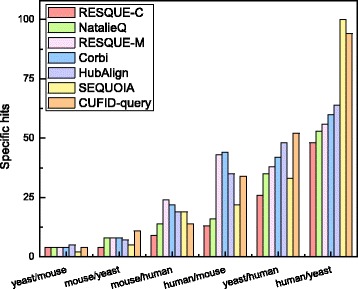
The number of specific hits for each network querying algorithm. Labels in the horizontal axis show the (query species)/(target PPI network) pairs

Figure 5 shows the number of specific hits for each network querying algorithm. Except the case comparing mouse and human, CUFID-query achieves a higher number of specific hits. When querying human complexes against the yeast PPI network, CUFID-query clearly outperforms competing methods. Although RESQUE-C achieves the largest number of hits and meaningful hits, it records the least number of specific hits because RESQUE-C includes a large number of less relevant nodes as we mentioned before. For overall 1242 test cases, CUFID-query achieves 15% more specific hits than the next best algorithm, SEQUOIA. Since the main goal of network querying is identifying the conserved subnetworks in the target network that are similar to the given query network, achieving a higher number of specific hits is more appropriate for the goal. These results mean that CUFID-query has a strong potential to accurately identify the known biological complex conserved in the target PPI network.

### Performance assessment: specificity

**Fig. 6:**
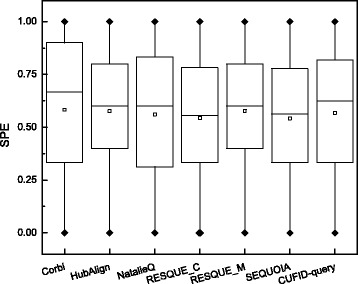
The specificity of the predictions made by different network querying algorithms. Each box plot shows the specificity of a given network querying algorithm. Note that the square corresponds to the mean value and the black diamonds indicate the outliers

**Fig. 7:**
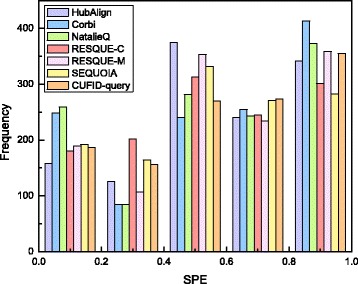
Histogram showing the specificity of each algorithm

We also checked the specificity of each network querying algorithm. Although the querying algorithm can identify the highly relevant subnetworks to the given query network, if it includes a larger number of less relevant nodes, it is difficult to exactly select the conserved subnetwork corresponding to a particular biological process. Figures 6 and 7 show a box plot and histogram of the specificity for each method. As shown in Fig. 6, although Corbi achieves the highest median value, the difference of the mean specificity for each method is negligible. Note that the median value for each method is as follows: Corbi (0.667), CUFID-query (0.625), HubAlign (0.6), NatalieQ (0.6), RESQUE-M (0.6) and RESQUE-C (0.556). CUFID-query still achieves higher specificity than HubAlign, NatalieQ, and RESQUE families. Interestingly, there are a number of outliers at either 0 or 1. Based on the box plot for the specificity, it is difficult to select the best algorithm in terms of the specificity because of the outliers. However, Fig. 7 shows that, although Corbi and NatalieQ can identify more querying results whose specificity is greater than 0.8, there are also a remarkably larger number of querying results whose specificity is smaller than 0.2. However, for CUFID-query, there are a relatively smaller number of querying results with low specificity, and there are a comparable number of querying results achieving fairly high specificity. This result indicates that querying results of the proposed method is comparably accurate and it includes a relatively smaller number of less relevant nodes.

Next, we also investigated the number of identified nodes and annotated nodes. As a network querying algorithm can be utilized to predict the functions of proteins in the identified network by transferring the knowledge about the functions of the querying network, identifying more annotated nodes would be advantageous for annotating functions of proteins in the target network (i.e., transferring the prior knowledge of the query network). Table 1 shows that RESQUE-C can identify a larger number of annotated nodes but the size of querying results is also relatively larger than the ones obtained by other methods. Hence, this causes the lowest percentage of annotated nodes for RESQUE-C. CUFID-query and Corbi show the similar percentages of annotated nodes, but CUFID-query can identify more annotated nodes than Corbi. This means that CUFID-query is more effective to accurately annotate protein functions in the novel biological complex (i.e., identified subnetwork in the target network).

**Table 1:** The number of identified nodes and the number of annotated nodes are summarized for different network querying algorithms and different query/target network pairs

	Human/mouse			Human/yeast		
	Annotated	Identified	% Annotated	Annotated	Identified	% Annotated
NatalieQ	1233	2454	0.502	1382	1753	0.788
Corbi	1357	2493	0.544	1305	1693	0.771
HubAlign	1343	2544	0.528	1692	2461	0.688
RESQUE-C	2019	4530	0.446	2735	3773	0.725
RESQUE-M	1395	2553	0.546	1669	2317	0.720
SEQUOIA	1799	3767	0.478	2807	3842	0.731
CUFID-query	1548	2946	0.525	2234	2969	0.752
	Mouse/human			Mouse/yeast		
	Annotated	Identified	% Annotated	Annotated	Identified	% Annotated
NatalieQ	223	366	0.609	161	193	0.834
Corbi	229	355	0.645	157	193	0.813
HubAlign	245	372	0.659	206	329	0.626
RESQUE-C	383	712	0.538	368	499	0.737
RESQUE-M	246	372	0.661	227	290	0.783
SEQUOIA	296	542	0.546	336	491	0.684
CUFID-query	277	447	0.620	274	397	0.690
	Yeast/human			Yeast/mouse		
	Annotated	Identified	% Annotated	Annotated	Identified	% Annotated
NatalieQ	767	1250	0.614	394	1223	0.322
Corbi	790	1230	0.642	424	1246	0.340
HubAlign	1003	1654	0.606	571	1683	0.339
RESQUE-C	1265	2490	0.508	772	2488	0.310
RESQUE-M	881	1574	0.560	543	1571	0.346
SEQUOIA	1171	2234	0.524	704	2337	0.301
CUFID-query	942	1507	0.625	531	1541	0.345
	Overall					
	Annotated		Identified		% Annotated	
NatalieQ	4160		7239		0.575	
Corbi	4262		7210		0.591	
HubAlign	5060		9043		0.560	
RESQUE-C	7542		14,492		0.520	
RESQUE-M	4961		8677		0.572	
SEQUOIA	7113		13,213		0.538	
CUFID-query	5806		9807		0.592	

### Performance assessment: computation time and stability of network querying algorithms

**Fig. 8:**
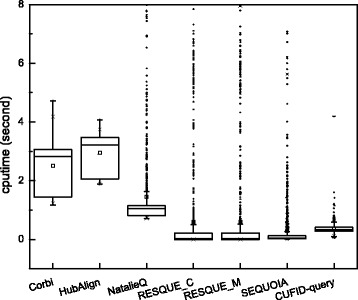
Computation time for each algorithm. Note that the black dots outside the whiskers correspond to outliers

To compare the computational complexity of each method, we compared the running time of each method. In this experiment, we tested all network querying algorithms using the same machine equipped with intel i7 dual core processor (2.9 GHz) and 16 GB memory. Figure 8 shows that CUFID-query, SEQUOIA, and RESQUE family are much faster than other algorithms, and NatalieQ records the next best in terms of the computation time. Interestingly, although the average of the computation time for NatalieQ, SEQUOIA and RESQUE family is very fast, there are a number of outliers. That is, they require unexpectedly longer time for network querying in some cases. These may depend on the topological structure of the query and target networks. That is, particular topological structures may require longer computation time for querying. Although CUFID-query also has outliers, the most cases complete the querying within a few seconds for all 1242 test cases. In addition to the computation time, NatalieQ fails to identify querying results for 71 queries among 1242 queries (i.e., NatalieQ can not find any matching nodes for 71 queries). This means NatalieQ may not be robust for a particular topological structure, but CUFID-query finds querying results for all 1242 queries and records a stable running time, where it implies the robustness of CUFID-query.

## Conclusions

In this paper, we propose a novel network querying algorithm, CUFID-query. We utilize the CUFID framework in order to estimate the correspondence (or biological relevance) between nodes in the query and large-scale target networks. In the CUFID framework, we first construct the integrated network by inserting pseudo-edges between nodes in the query and target networks, and we design a random walker whose random transition through a pseudo-edge is proportional to both node and topological similarities. Hence, we can effectively estimate the node correspondence by measuring a steady-state network flow across the pseudo-edges with a reduced computational cost. Based on the estimated node correspondence scores through the CUFID framework, CUFID-query identifies the seed network (i.e., high correspondence region in the target network). Then, we iteratively extend the seed network by adding a selected node, based on the association probability and the conductance minimization criterion. Finally, in case that the seed-and-extension approach may include irrelevant nodes, we remove less relevant nodes based on the personalized PageRank vector for the induced network. Through an extensive performance evaluation using 1242 known biological complexes and large-scale PPI networks, we have shown that CUFID-query leads to accurate and functionally consistent querying results. In this study, we have verified that the CUFID framework is effective to compare biological networks with significant different sizes. Additionally, several algorithms require significantly longer computation time to identify conserved biological networks. Accommodating structural variations between conserved networks and insufficient information for pairwise node similarity (i.e., protein homology) would be the major reasons, and this should be taken into account to develop more advanced network querying algorithms in the future. More importantly, since there is no gold standard benchmark dataset that can be used to assess and compare the performance of different network querying algorithms, it is important to develop standard performance assessment methods based on comprehensive and balanced benchmark datasets for network querying.
